# A nuclear factor-binding domain in the 5'-untranslated region of the *amyloid precursor protein *promoter: Implications for the regulation of gene expression

**DOI:** 10.1186/1756-0500-3-4

**Published:** 2010-01-12

**Authors:** Alexander A Vostrov, Michael J Taheny, Nerik Izkhakov, Wolfgang W Quitschke

**Affiliations:** 1 Department of Psychiatry and Behavioral Science, State University of New York at Stony Brook, Stony Brook, NY 11794-8101, USA

## Abstract

**Background:**

The extracellular deposition of aggregated amyloid β-protein is a neuropathological manifestation of Alzheimer disease and Down syndrome. The Amyloid β-protein is derived from a group of larger differentially spliced proteins, the amyloid protein precursors (APP). Data suggests that the level of *APP *gene expression could contribute to the pathological processes leading to amyloid depositions.

**Findings:**

The 5' untranslated region (UTR) of the *APP *gene, encompassing 147 base pairs between the transcriptional (+1) and the translational start site, was examined for its role in *APP *expression. Deletions close to the transcriptional start site reduced expression from the *APP *promoter in part by transcriptional mechanisms. However, deletions between position +50 and +104 had no effect on transcriptional activity while significantly reducing overall expression from the promoter. A nuclear factor-binding domain designated as DAPB was identified between position +72 and +115 of the 5'-*APP*-UTR. The binding-recognition sequence was localized between position +96 and +105. The same mutations that eliminated factor-binding also reduced expression from the *APP *promoter while having no effect on *in vitro *transcription or the RNA levels transcribed from transfected constructs.

**Conclusions:**

A nuclear factor-binding domain designated as DAPB was identified in the 5'-UTR of the *APP *gene. Elimination of factor-binding correlated with an overall decline in expression from the *APP *promoter while *in vitro *transcription and the total amount of *in vivo *transcribed RNA remained unaffected. This suggests that the binding-factor may have a function in post-transcriptional regulation, including nuclear export of mRNA.

## Background

A neuropathological manifestation of Alzheimer disease and Down syndrome is the extracellular deposition of aggregated amyloid β-protein [1-3]. The Amyloid β-protein is derived from a group of larger differentially spliced proteins, the amyloid protein precursors (APP) [4]. There are three copies of the *APP *gene in Down syndrome and the level of *APP *transcript in the brains of afflicted individuals is increased about 4.5-fold [5]. A chimeric mouse model of Down syndrome shows increased *APP *transcription associated with cholinergic neuron degeneration [6]. Promoter mutations that increase the expression of APP are associated with the development of Alzheimer disease and a duplication of the *APP *gene locus causes autosomal dominant early-onset Alzheimer disease [7,8]. These observations suggest that the level of expression of the *APP *gene could contribute to the pathological processes leading to amyloid depositions. The 5'-untranslated region (UTR) was here examined for its role in *APP *expression.

## Results and discussion

### Deleting 5'-APP-UTR sequences between position +10 and +104 decreases expression from the APP promoter

**Figure 1:**
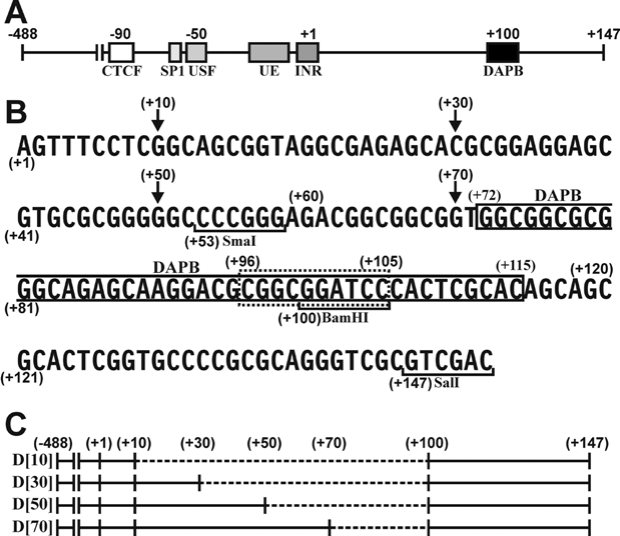
**Structure of the *APP *promoter and the 5' UTR**. (A) Schematic representation of the *APP *promoter with boxes indicating locations of regulatory regions and the DAPB domain discussed in the text. (B) The sequence of the 5'-*APP*-UTR from position +1 to +147. The translational start site (ATG) would follow position +147 in the native *APP *gene. Also shown are deletions at position +10, +30, +50, and +70 (arrows) and pertinent nucleotide positions as discussed in the text. Brackets show relevant restriction sites, of which SalI is part of the plasmid polylinker region. The sequence defining DNase I protected domain DAPB (solid lines) and the sequence recognized by the binding factor (dotted lines) are indicated by boxes. (C) Schematic representation of internal deletions D [10]-D [70] within the APP [147] fragment. Deleted sequences are indicated by dotted lines.

**Figure 2:**
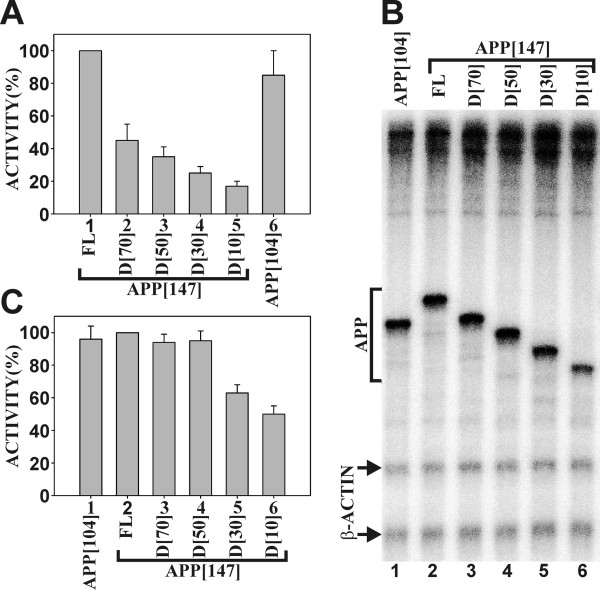
**Expression from the *APP *promoter with deletions within the 5'-*APP*-UTR**. (A) Expression from the full-length *APP *promoter and internal 5'-*APP*-UTR deletions by transient transfection in HeLa cells. The results are presented as CAT activities relative to the wild type APP [147] construct, which was assigned the value of 100% (column 1). Columns 2-5 show internal deletions D [70]-D [10] within APP [147] and column 6 shows the APP [104] construct that terminates at position +104. The results represent the average values of at least ten separate transfections with standard deviations (error bars). (B) Run-off *in vitro *transcription from promoter construct APP [104] (lane 1), APP [147] (lane 2) and sequential deletions D [70]-D [10] (lanes 3-6). The bracket delineates fragments transcribed from the *APP *promoter. Arrows indicate the two transcripts originating from the *β-actin *promoter. (C) Quantitation of transcription reactions illustrated in B. The *APP *transcripts were normalized to identical *β-actin *transcripts and the full-length (FL) APP [147] construct was assigned the value of 100% (column 2). Results represent the average of four independent experiments with standard deviations (error bars).

Four incremental internal deletions within 5'-*APP*-UTR construct APP [147], designated as D [10], D [30], D [50], and D [70] were analyzed by transient transfection (Fig. 1B and 1C). The deletions resulted in stepwise reductions of CAT activity from 48% (D [70]) to 18% (D [10]) of the wild type value (Fig. 2A, columns 1-5).

The deletion constructs were further analyzed by run-off *in vitro *transcription. The constructs with deletions in the 5'-*APP*-UTR region resulted in run-off fragments of correspondingly smaller size (Fig. 2B, lanes 1-6). Transcription from deletions D [30] and D [10] was reduced to 68% and 50% of the wild type value, respectively (Fig. 2C, columns 1-6). Since the deleted sequences in D [30] and D [10] are close to the transcriptional start site, they may be considered components of the core promoter. Downstream core promoter sequences such as DPE [9], DCE [10] or MTE [11] that are located within the first 35 nucleotides 3' to the transcriptional start site are often required for efficient transcription. However, the downstream region of the *APP *promoter does not match the consensus sequences for any of these elements. This may imply the existence of a non-conventional proximal downstream element in the *APP *core promoter. The remaining deletions D [50] and D [70] had no effect on *in vitro *transcriptional activity, while they did diminish *in vivo *expression from the *APP *promoter (Fig. 2A and 2C). In contrast, a 3'-deletion terminating at position +104 (APP [104]) had no significant effect on *APP *promoter expression (Fig. 2A-C).

### A nuclear factor displays sequence specific binding to the 5'-APP-UTR

**Figure 3:**
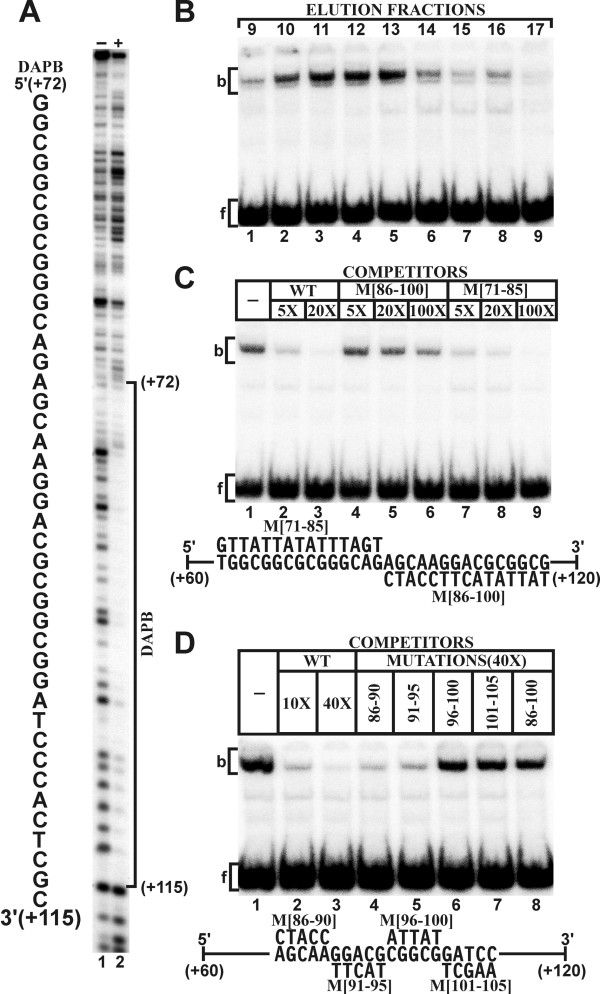
**Characterization of the DAPB binding domain in the 5'-*APP*-UTR**. (A) DNase I footprinting of a wild type *APP *promoter fragment extending from position -40 including the UTR to position +147 (lanes 1 and 6). The coding strand was 5' end-labeled with [^32^P] and the resulting fragment was digested with DNase I either in the presence (+) or absence (-) of nuclear extract. Brackets delineate the position of DNase I protected domain DAPB from position +72 to +115 as discussed in the text. (B) Mobility shift electrophoresis with elution fractions from SP ion exchange chromatography of whole HeLa cell nuclear extract and a [^32^P] 5' end-labeled double stranded oligonucleotide containing the 5'-*APP*-UTR sequence from position +60 to +120 [60-120]. The binding complex eluted predominantly in fractions 11-13 (lanes 3-5). The binding complex (b) and the free oligonucleotide (f) are indicated by brackets throughout the figure. (C) Mobility shift competition with the 5'-*APP*-UTR fragment [60-120] as a labeled probe: Mobility shift without competitor (lane 1); mobility shift with a 5-fold (lane 2) and 20-fold (lane 3) molar excess of unlabeled wild type [60-120] sequence; competition with 5-fold (lane 4), 20-fold (lane 5), and 100-fold (lane 6) excess of unlabeled oligonucleotide containing transverse mutations from position +86 to +100 (M [86-100]) within the [60-120] fragment (lower panel); competition with a 5-fold (lane 7), 20-fold (lane 8), and 100-fold (lane 9) excess of unlabeled oligonucleotide containing transverse mutations from position +71 to +85 (M [81-85]). (D) Mobility shift competition with the [60-120] fragment as a labeled probe (lane 1). Self-competition with a 10-fold (lane 2) and 40-fold (lane 3) excess of wild type [60-120] sequence and competition with a 40-fold unlabeled excess of mutations M [86-90] (lane 4), M [91-95] (lane 5), M [96-100] (lane 6), M [101-105] (lane 7), and M [86-100] (lane 8).

The *APP *sequence from position -40 to +147 was analyzed by DNase I footprinting with HeLa cell nuclear extract (Fig. 3A). A prominent DNase I protected domain that extended from position +72 to +115 was designated as DAPB.

The factors that interact with the DAPB sequence were further examined by mobility shift electrophoresis with nuclear extract fractionated on SP-Sepharose. The major DNA binding activity eluted in fractions 10-14 (Fig. 3B, lanes 2-6). Fractions 11 and 12 were combined and used for further experiments. The binding complex was effectively competed with 5- and 20-fold molar excesses of unlabeled wild type [60-120] sequence (Fig. 3C, lanes 1-3). Transverse mutations within the [60-120] double stranded oligonucleotide were introduced from positions +71 to +85 (M [71-85]) and +86 to +100 (M [86-100]). Factor-binding was not effectively competed with a 100-fold molar excess of unlabeled M [86-100] fragment (Fig. 3C, lanes 4-6), whereas the M [71-85] fragment competed the binding with a five-fold molar excess (Fig. 3C, lanes 7-9). Additional mobility shift competitions with 40-fold molar excesses of successive transverse mutations M [86-90]-M [101-105] (Fig. 3D, lanes 4-7) showed that only M [96-100] and M [101-105] did not compete (Fig. 3D, lanes 6-7). These results suggest that the primary binding recognition sequence is located between position +96 and +105.

### Expression from the APP promoter is reduced in mutations that eliminate nuclear factor-binding to DAPB

**Figure 4:**
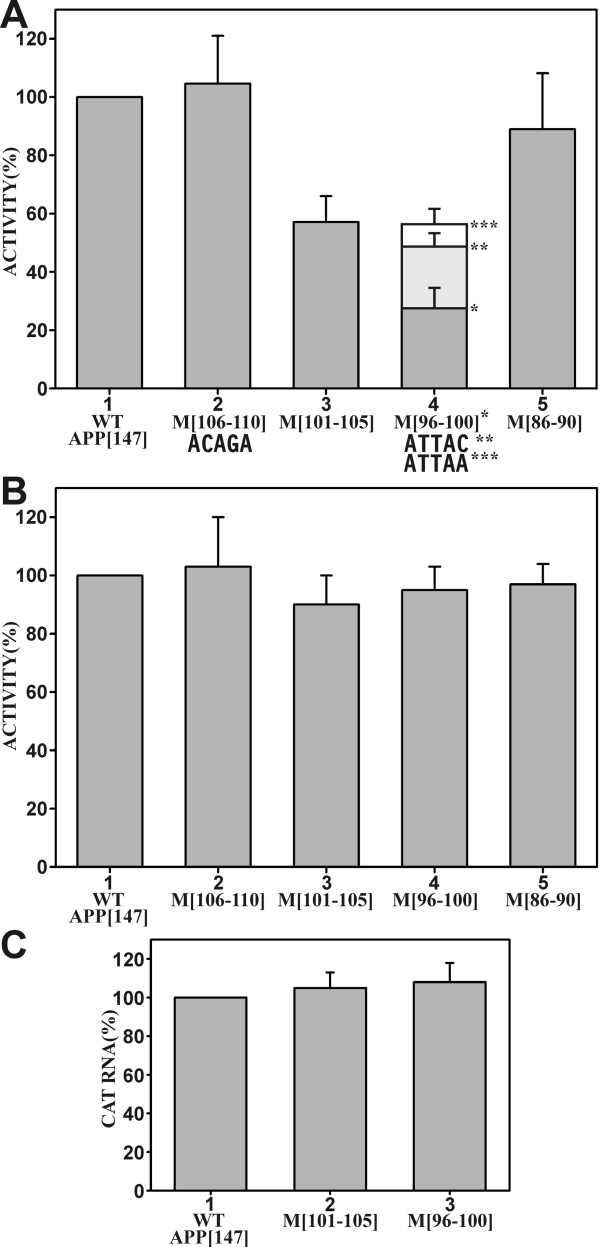
**Expression from the *APP *promoter with mutations within the 5'-*APP*-UTR**. (A) Transient transfection in HeLa cells. The activities of consecutive 5-base-pair block mutations M [106-110]-M [86-90](columns 2-5) are presented as CAT activities relative to the wild type APP [147] construct (column 1), which was assigned the value of 100%. In addition to the original M [96-100] mutation (column 4, *) [see Fig. 3D for sequence], the activities of two additional mutations (column 4, ** and ***) that removed the ATG sequence were investigated. The results represent the average values of at least ten separate transfections with standard deviations (error bars). (B) Run-off *in vitro *transcription in HeLa cell nuclear extract with the same designations as in A. (C) *CAT *RNA levels in HeLa cells transfected with constructs APP [147] WT (column 1), M [101-105] (column 2), and M [96-100] (column 3) were determined by quantitative RT-PCR. The wild type APP [147] construct (column 1) was assigned the value of 100% and the results represent the average of four independent experiments with standard deviations (error bars).

The same mutations that eliminated nuclear factor-binding to the DAPB site also decreased expression from the respective APP [147] promoter constructs by transient transfection. Transverse mutation M [101-105] resulted in a decrease to 55% of the wild type value, whereas mutation M [96-100] decreased expression levels to 28% (Fig. 4A). Additional mutations at position +100 within construct M [96-100] were also investigated, since the original transverse mutation (Fig. 3D) generated an additional translational start site (ATG) within the 5'-*APP*-UTR, which may have interfered with translational activity. This additional start site was eliminated by replacing the terminal T residue with either C or A residues (Fig. 4A, column 4). Indeed, in those constructs the activity from the *APP *promoter was reduced to only 50% of the wild type value, which was consistent with the results obtained with the M [101-105] construct (Fig. 4A, column 3) and internal deletion D [70](Fig. 2A, column 2). This strongly suggests that this effect is dependent on nuclear factor-binding to the DAPB site. None of the mutations significantly affected *in vitro *transcriptional activity or the level of *CAT *RNA in transfected cells (Fig. 4B,C).

The DAPB mediated reduction in *APP *promoter expression *in vivo *is therefore likely due to post-transcriptional events. Indeed, an analysis employing a combination of RNA fractionation and tiling arrays identified post-transcriptionally regulated *APP *expression in HeLa S3 and SK-N-AS cells [12]. Several other studies have implicated the 5'-*APP*-UTR in post-transcriptional gene regulation [13-18]. For example, it was shown that *APP *expression is regulated by an iron response element (IRE) and an IL-1 acute box responsive domain downstream from the IRE at position +101 to +146 [13,15]. However, binding of both IRP and IL-1 to RNA occurs in the cytoplasm and would thus represent later stages of the translational process. Since the binding of DAPB occurs on double stranded DNA with nuclear extract, it is unclear how the binding of IRP and IL-1 relates to the functional features of the 5'-*APP*-UTR described here. It has also been demonstrated that APP synthesis is regulated through an internal ribosome entry site (IRES) [19]. However, this IRES is located within the first 50 nucleotides of the 5'-*APP*-UTR and therefore outside of the DAPB binding domain. In additional studies, a negative thyroid hormone response element (nTRE) between position +80 and +96 of the 5'-*APP*-UTR that functions in conjunction with an adjacent SP1 binding site was described [20,21]. However, Mobility shift competition with oligonucleotides containing the direct repeat TRE sequence (TRE-DR4) [22] showed no competition with the APP [60-120] element for DAPB binding (not shown). Furthermore, the transverse mutations that eliminate DAPB complex formation on the [60-120] fragment are actually located outside the postulated TR recognition sequence and the SP1 binding site. The observation that DAPB binding was non-specifically competed by RNA (not shown) may suggest a role in nuclear export of nascent mRNA.

## Conclusion

The 5'-*APP*-UTR was analyzed for its effect on promoter expression by transient transfection. Deletions close to the transcriptional start site reduced expression from the *APP *promoter in part by transcriptional mechanisms. Deletions between position +104 and +50 had no effect on transcriptional activity while significantly reducing overall expression from the promoter. A nuclear factor-binding domain designated as DAPB from position +72 to +115 within the 5'-*APP*-UTR was identified with a recognition sequence located between position +96 and +105. The same mutations that eliminated factor-binding also reduced expression from the *APP *promoter while having no effect on *in vitro *transcription or RNA levels transcribed from transfected constructs. These results suggest that the nuclear binding-factor exert its effect through post-transcriptional mechanism. The nuclear localization of the binding factor and its affinity for RNA may suggest a function in RNA transport. A review of the literature of related studies suggested that the DAPB binding-factor is distinct from those previously described.

## Materials and methods

### Cell cultures, promoter constructs, and enzyme assays

Cell cultures, transient transfections, enzyme assays, and oligonucleotide preparations were carried out as previously described [23,24].

Plasmid pCAT2bGAL [25] contains the bacterial genes *CAT *and *LacZ*, which are both transcribed in the same direction. The *LacZ *gene is transcribed from the *β-actin *promoter and serves as an internal control for experimental variations. The polycloning site on the 5'-end of the *CAT *transcriptional unit was the recipient for the various *APP *promoter constructs. The 5'-end of the full-length *APP *promoter (APP [147]) construct extended from position -488 upstream from the transcriptional start site (+1)[24]. This upstream promoter region contains all nuclear factor-binding sites relevant for efficient *APP *expression (Fig. 1A). The 3'-end extended to position +147, which in the endogenous *APP *gene transcript would have been followed by the translational start site (Fig. 1B). Mutations and deletions within wild type APP [147] were generated by *in vitro *mutagenesis [26,27].

### Nuclear extracts, mobility shift electrophoresis, and DNase I footprinting

Nuclear extracts were prepared from HeLa cells grown in suspension to a density of 5-8 × 10^5 ^cells/ml [23,24]. The final protein concentration in extracts was 10-15 mg/ml in buffer D [23,24]. For fractionation, a 10 ml SP-Sepharose Fast Flow column (GE Healthcare) was equilibrated with buffer D. Ten ml of nuclear extract was loaded on the column, washed with 15 ml of buffer D, and eluted with a 50 ml linear gradient of 200 mM-700 mM KCl in buffer D. Fractions of 2 ml were collected and analyzed by mobility shift electrophoresis.

For mobility shift electrophoresis, binding reactions were assembled in a total reaction volume of 16 μl and processed as described [23]. In competition assays, labeled and unlabeled oligonucleotides were premixed at indicated molar ratios before adding them to the binding reaction.

DNase I footprinting was conducted on *APP *DNA fragments extending from position -40 to +147. The fragments were amplified by PCR with the [^32^P] end-labeled reverse primer complementary to the 5'-*APP*-UTR from position +142 to +113 and unlabeled forward primer from position -40 to -29 [24]. The resulting double stranded DNA fragment was purified by polyacrylamide gel electrophoresis in 0.5 × TBE. Binding reactions were preincubated with 8 μl of HeLa cell nuclear extract and 50 × 10^3 ^cpm of labeled fragment under the same conditions as described above for mobility shift electrophoresis, except that the total reaction volume was adjusted to 32 μl, and processed as described [23].

### Run-off in vitro transcription

Plasmid pCAT2bGAL containing the wild type or mutated APP [147] constructs were digested with restriction enzymes EcoRI and Acc65I. *In vitro *transcription reactions were assembled in a volume of 8 μl containing 2.5 μl of nuclear extract, 0.5 μl (1 μg) of plasmid template, and 5 μl of a buffer containing 40 mM Hepes, pH 7.6, 75 mM KCl, 2 mM MgSO_4_, 0.1 mM EDTA, 1 mM DTT, and 10% glycerol. Thereafter, 0.5 μl of 10 mM GTP, 10 mM CTP, 3.3 mM ATP, 3 mM UTP and 0.3-0.7 μl (10^6 ^cpm) of α-[^32^P]-UTP (PerkinElmer) was added and reactions were incubated at 30°C for 30 min. Transcribed RNA was purified with the RNeasy (Qiagen) RNA isolation kit.

### Quantitative RT-PCR of RNA from transfected cells

Total RNA was prepared from transfected cells with the RNeasy Mini kit, digested with 10 units of RNase free DNase I (Roche), and repurified. One μl of purified RNA (0.3-0.6 μg) was combined in 10 μl annealing buffer containing 300 mM NaCl, 10 mM Tris, pH 7.5, 1 mM EDTA with 10 pmol each of primers Gal-R (5'-GGTTACGTTGGTGTAGATGGGCG) complementary to the *LacZ *transcript, and CAT-R (5'-TGAGCATTCATCAGGCGGGC) complementary to the *CAT *transcript. The solution was heated to 99°C for 5 minutes, incubated at 61°C for 5 min, at 57°C for 10 min, and at 48°C for 5 min. To each annealing mixture was added 40 μl of a reverse transcriptase reaction mixture containing 12.5 mM Tris, pH 8.0, 10 mM MgCl_2_, 12.5 mM DTT, 1.25 mM each of the dNTPs, 20 units of AMV reverse transcriptase (CHIMERx), and 10 units of RNasin (Invitrogen). The reaction was continued at 48°C for 90 min.

PCR amplification of 4 μl aliquots of the reverse transcription reaction products was assembled in 40 μl of Mg+ PCR buffer (Roche) supplemented with 0.2 mM of each dNTP, 0.4 pmol each of 5' end-labeled (50-100 × 10^3 ^cpm) CAT-R, Gal-R, unlabeled CAT-F (5'-TTTCAGGAGCTAAGGAAGCTAAAATGGAG) and Gal-F (5'-CTGAGCCGCGATATTGCCCAG) primers. Amplification was carried out for 18 cycles. PCR products were separated on 5% polyacrylamide gels. The amount of *CAT *RT-PCR product was normalized to the amount of *LacZ *RT-PCR product. To establish that the RT-PCR procedure was quantitative, different amounts of RNA and different numbers of PCR cycles were used. Lack of plasmid contamination in RNA preparations was confirmed by amplifying aliquots of reverse transcription reaction mixture prior to reverse transcription.

## Abbreviations

APP: amyloid precursor protein; UTR: untranslated region; CAT: chloramphenicol acetyltransferase; DTT: dithiothreitol; CHAPS: 3 [(3-cholamidopropyl)dimethylammonio]-propanesulfonic acid; Hepes: N-2-Hydroxyethylpiperazine-N'-2-ethanesulfonic acid; Tris: Tris-(hydoxymethyl)aminomethane; EDTA: ethylenediamine tetraacetic acid; TBE: tris-borate-EDTA; cpm: counts per minute; RT: reverse transcriptase; PCR: polymerase chain reaction; IRE: iron response element; IRP: iron regulatory protein; TRE: thyroxin response element; C: cytidine; T: thymidine; A: adenosine

## Competing interests

The authors declare that they have no competing interests.

## Authors' contributions

MT, WQ, and NI carried out molecular cloning, transfections, and *in vitro *mutagenesis. AV performed DNase I footprinting, mobility shift electrophoresis, and *in vitro *transcription. All authors contributed to data analysis. WQ and AV wrote the manuscript. All authors read and approved the final manuscript.
